# Supplementary material for: Reconciling Mining with the Conservation of Cave Biodiversity: A Quantitative Baseline to Help Establish Conservation Priorities
Source: PLoS One. 2016 Dec 20;11(12):e0168348. doi: 10.1371/journal.pone.0168348 (PMC5173368; doi:10.1371/journal.pone.0168348)
Supplement: S1 Dataset — (ZIP) [file pone.0168348.s002.zip › Taxa/Serra Norte/SN_2013/N5S/Lista N5S-54.pdf]

## CAVIDADE N5S-0054

| Classe | Ordem | Fam/Outros | Gên/Outros | Espécie | S-U | U-S | Zon. |
|---|---|---|---|---|---|---|---|
| Arachnida | Amblypygi | Phrynidae | *Heterophrynus* | *longicornis* | | 1 | E |
| Arachnida | Araneae | Araneidae | *Alpaida* | aff. *negro* | | X | E |
| Arachnida | Araneae | Caponiidae | | jovem | X | | E |
| Arachnida | Araneae | Corinnidae | | jovem | | 2 | E |
| Arachnida | Araneae | Ctenidae | | jovem | 1 | | E |
| Arachnida | Araneae | Drymusidae | | jovem | X | X | E |
| Arachnida | Araneae | Pholcidae | | jovem | | X | E |
| Arachnida | Araneae | Pisauridae | | jovem | | 1 | E |
| Arachnida | Araneae | Salticidae | | jovem | X | X | E |
| Arachnida | Araneae | Scytodidae | *Scytodes* | jovem | | 2 | E |
| Arachnida | Araneae | Theridiidae | | jovem | X | | E |
| Arachnida | Araneae | Theridiosomatidae | *Plato* | sp.1 | X | X | E |
| Arachnida | Opiliones | | | jovem | | 1 | E |
| Arachnida | Pseudoscorpiones | Chthoniidae | *Pseudochthonius* | sp.1 | X | X | E |
| Chilopoda | Scutigeromorpha | Pselliodidae | *Sphendononema* | jovem | 1 | | E |
| Entognatha | Collembola | Entomobryidae | | sp.1 | X | | E |
| Entognatha | Collembola | Entomobryidae | | sp.9 | X | | E |
| Entognatha | Collembola | Paronellidae | | sp.1 | | X | E |
| Insecta | Blattodea | Blattellidae | | sp.1 | 4 | 6 | E |
| Insecta | Blattodea | Polyphagidae | | sp.1 | | 3 | E |
| Insecta | Coleoptera | Scydmaenidae | | sp.1 | | X | E |
| Insecta | Coleoptera | Staphylinidae | | sp.1 | | X | E |
| Insecta | Diptera | Culicidae | *Culex* | sp. | X | | E |
| Insecta | Diptera | Sciaridae | | sp. | | X | E |
| Insecta | Diptera | Tipulidae | Tipulinae | sp. | | X | E |
| Insecta | Homoptera | Cixiidae | | jovem | | X | E |
| Insecta | Hymenoptera | Diapriidae | | sp.3 | | X | E |
| Insecta | Hymenoptera | Formicidae | *Camponotus* | sp.2 | X | | E |
| Insecta | Hymenoptera | Formicidae | *Odontomachus* | sp.1 | | X | E |
| Insecta | Hymenoptera | Formicidae | *Paratrechina* | sp.1 | X | | E |
| Insecta | Hymenoptera | Formicidae | *Paratrechina* | sp.2 | X | | E |
| Insecta | Hymenoptera | Formicidae | *Solenopsis* | sp.1 | | X | E |
| Insecta | Isoptera | Termitidae | *Subulitermes* | sp. | X | X | E |
| Insecta | Lepidoptera | Gelechioidea | | sp. | X | X | E |
| Insecta | Orthoptera | Phalangopsidae | *Paraclodes* | sp. | 3 | | E |
| Insecta | Psocoptera | Epipsocidae | *Epipsocus* | sp.1 | | X | E |
| Insecta | Psocoptera | Manicapsocidae | *Nothoentomum* | sp.1 | | X | E |
| Insecta | Psocoptera | Ptiloneuridae | *Ptiloneura* | sp.2 | X | | E |
| Insecta | Thysanoptera | Phlaeothripidae | | sp.1 | X | | E |
| Insecta | Thysanura | Ateluridae | | sp.1 | X | X | E |
| Insecta | Thysanura | Nicoletiidae | | sp.1 | X | X | E |
| Malacostraca | Isopoda | Philosciidae | | sp.1 | | X | E |
| Amphibia | Anura | Strabomantidae | *Pristimantis* | cf. *fenestratus* | 2 | 3 | E |
